# Supplementary material for: The two extremes of Hansen’s disease—Different manifestations of leprosy and their biological consequences in an Avar Age (late 7th century CE) osteoarchaeological series of the Duna-Tisza Interfluve (Kiskundorozsma–Daruhalom-dűlő II, Hungary)
Source: PLoS One. 2022 Jun 23;17(6):e0265416. doi: 10.1371/journal.pone.0265416 (PMC9223331; doi:10.1371/journal.pone.0265416)
Supplement: S1 Text — (PDF) [file pone.0265416.s001.pdf]

S1 Text: The Avar Khaganate in the Carpathian Basin between the 6th and 8th centuries CE.

Arriving together with other Eastern European nomads from the Eurasian steppe, the Inner Asian Avars united the local peoples and communities of the Carpathian Basin (568 CE). The rich Avar archaeological record comprises the material known from roughly 100,000 burials and 600 settlements. The concentration of high-status burials lavishly furnished with weapons and gold artefacts in the Duna-Tisza Interfluve suggests that the Avar power centre lay in this region during the 6th and 8th centuries CE. The highest-ranking members of the early Avar elite (the leaders of the Avar polity and the khagan's military retinue) were buried with gold- and silver-decorated weapons and belts, various insignia, and valuable prestige objects. The Eastern European nomads settling in eastern Hungary (Trans-Tisza region) can be characterised by solitary burials or small burial grounds, the deposition of partial or complete sacrificial animals, and ledge or niche graves. The western Carpathian Basin (the former Roman province of Pannonia) was a border zone in the Avar Khaganate, populated by communities with different cultural traditions, such as the remnants of the Romanised population and Germanic groups. The Avar elite used its power to cement the peoples of the Carpathian Basin and integrate them into the Avar Khaganate in the 7th century CE and to ensure the powerful political position of the Avars in Europe. Following the ultimate failure of the wars against Byzantium (626 CE), internal strife broke out in the Avar Khaganate, and the economic basis of the Avar elite shifted to agrarian production in rural villages. Material culture underwent a transformation and became more uniform and standardised. The late 7th century CE saw the arrival of new population groups from the east, and although the size of the territories controlled by the Avars increased, there are few indications of an active foreign policy. The "speed" and success of acculturation is reflected by the emergence of a uniform material culture across the Avar Khaganate in the 8th century CE (e.g., belts decorated with Mediterranean and Eurasian motifs and patterns), expressing the unity of the Avar political organisation. Owing to their eastern-type social organisation and pagan beliefs, the Avars were unable to fit into Christian Europe – their social and political system grew weak, and they were unable to withstand the attacks launched by the Franks and the Bulgars.
