# Supplementary material for: The two extremes of Hansen’s disease—Different manifestations of leprosy and their biological consequences in an Avar Age (late 7th century CE) osteoarchaeological series of the Duna-Tisza Interfluve (Kiskundorozsma–Daruhalom-dűlő II, Hungary)
Source: PLoS One. 2022 Jun 23;17(6):e0265416. doi: 10.1371/journal.pone.0265416 (PMC9223331; doi:10.1371/journal.pone.0265416)
Supplement: S2 Text — (PDF) [file pone.0265416.s002.pdf]

S2 Text: Components of the rhinomaxillary syndrome in leprosy.

The so-called rhinomaxillary syndrome involves the anterior nasal spine, the inferior and lateral margins of the pyriform aperture, the alveolar and palatine processes of the maxilla, and the intranasal bony structures, such as the bony nasal septum and the nasal conchae [1-9]. These anatomical components can occur alone or in any combination with each other [3]. The rhinomaxillary syndrome consists of the following lesions appearing in the aforementioned anatomical regions:

1) Surface pitting, progressive resorption, and eventual disappearance of the anterior nasal spine. The initial loss of the cortical bone leads to exposure of the cancellous bone. Following the disappearance of the anterior nasal spine, at its original base, the exposed bone trabeculae can be covered as a result of cortical remodelling [1-6,9];

2) Progressive, bilaterally symmetrical resorption and rounding/remodelling of the inferior and lateral margins of the pyriform aperture that result in inferior widening of the anterior bony opening of the nasal cavity. The pathological process appears to involve the inferior third to half of the pyriform aperture. At the normally less sharply defined inferior margins, there is surface pitting and progressive resorption that leads to exposure of the cancellous bone. Later in the pathogenesis, the exposed bone trabeculae can be covered as a result of cortical remodelling. At the normally sharply defined lateral margins, there is smooth remodelling without exposure of the cancellous bone as the cortical covering is maintained. Usually, there is no surface pitting in this area [1-6,9];

3) Progressive resorption, recession, and remodelling of the maxillary alveolar process that is restricted largely to the premaxilla. The pathological process begins centrally at the prosthion and extends bilaterally, symmetrically to the alveoli of the central and lateral upper incisors; whilst proceeding laterally, it also extends superiorly. The smooth, progressive resorption firstly affects the anterior alveolar walls and later the posterior ones, and results in loosening and ultimate *ante-mortem* loss of the incisors devoid of their alveoli. Although there is an extensive bone resorption in the premaxillary region, the thickness of the cortical bone is maintained, presumably via endosteal bone deposition. Besides the above-mentioned bony changes, pitting indicative of superficial inflammation can develop on the anterior surface of the maxillary alveolar process [1-6,9];

4) Inflammatory changes on the nasal and/or oral surfaces of the maxillary palatine process in the form of pitting, erosion, thinning, and ultimate perforation of the hard palate (usually in the median or paramedian position) that can sometimes be accompanied by subperiosteal new bone formation on the affected bone surface(s). At the beginning of the pathological process, there is a generalised fine pitting that becomes more gross and confluent over time. Besides surface pitting, discrete, roughly circular, shallow, single or multiple erosive lesions develop that are rarely larger than 0.5 cm in diameter. The formation of the above-mentioned lesions leads to thinning and eventual perforation of the hard palate [1-6,9];

5) Inflammatory changes of the bony nasal septum and/or nasal conchae (predominantly the inferior ones) in the form of pitting, progressive resorption, and ultimate disappearance of one or more of these intranasal structures [1-6,9].

REFERENCES

1) Møller-Christensen V, Bakke SN, Melsom RS, Waaler AE. Changes in the anterior nasal spine and the alveolar process of the maxillary bone in leprosy. Int J Lepr. 1952;20(3): 335-340.
2) Møller-Christensen V. Changes in the anterior nasal spine and the alveolar process of the maxillae in leprosy: A clinical examination. Int J Lepr. 1974;42(4): 431-435.
3) Andersen JG, Manchester K. The rhinomaxillary syndrome in leprosy: A clinical, radiological, and palaeopathological study. Int J Osteoarchaeol. 1992;2(2): 121-129. doi: 10.1002/oa.1390020204
4) Aufderheide AC, Rodríguez-Martín C. The Cambridge encyclopedia of human paleopathology. Cambridge, UK: Cambridge University Press; 1998.
5) Crane-Kramer GMM. The paleoepidemiological examination of treponemal infection and leprosy in medieval populations from northern Europe. PhD thesis, University of Calgary (Calgary, AB, Canada). 2000. doi: 10.11575/PRISM/12209
6) Ortner DJ. Infectious diseases: Tuberculosis and leprosy. In: Ortner DJ, editor. Identification of pathological conditions in human skeletal remains. San Diego, CA, USA: Academic Press; 2003. pp. 227-271.
7) Kasai N, Kondo O, Suzuki K, Aoki Y, Ishii N, Goto M. Quantitative evaluation of maxillary bone deformation by computed tomography in patients with leprosy. PLoS Negl Trop Dis. 2018;12(3): e0006341. doi: 10.1371/ journal.pntd.0006341

8) Lewis M. Infectious diseases II: Infections of specific origin. In: Paleopathology of children. Identification of pathological conditions in the human skeletal remains of non-adults. London, UK: Academic Press; 2018. pp. 151-192. doi: 10.1016/B978-0-12-410402-0.00007-2

9) Roberts CA, Buikstra JE. Bakterial infections. In: Buikstra JE, editor. Ortner's Identification of pathological conditions in human skeletal remains. San Diego, CA, USA: Academic Press; 2019. pp. 321-439. doi: 10.1016/B978-0-12-809738-0.00011-9
