# Supplementary material for: The two extremes of Hansen’s disease—Different manifestations of leprosy and their biological consequences in an Avar Age (late 7th century CE) osteoarchaeological series of the Duna-Tisza Interfluve (Kiskundorozsma–Daruhalom-dűlő II, Hungary)
Source: PLoS One. 2022 Jun 23;17(6):e0265416. doi: 10.1371/journal.pone.0265416 (PMC9223331; doi:10.1371/journal.pone.0265416)
Supplement: S3 Text — (PDF) [file pone.0265416.s003.pdf]

S3 Text: Differential diagnoses of the rhinomaxillary bony changes indicative of leprosy that were observed in KD271.

The most relevant differential diagnoses of the rhinomaxillary skeletal lesions indicative of leprosy that were observed in **KD271** are bacterial granulomatous infections other than leprosy (e.g., treponematoses, tuberculosis, and actinomycosis), systemic fungal infections (e.g., aspergillosis and mucormycosis), and sarcoidosis [1-4].

From the four medical conditions collectively referred to as treponematoses, pinta, bejel, yaws, and syphilis, the three latter ones can affect the bones in their advanced stages [1,4-6]. Bejel, yaws, and syphilis are caused by three different subspecies of the bacterium *Treponema pallidum* [6-7]. As pinta never involves the skeleton [1,4-6], it can be ruled out as a diagnostic option in **KD271**. Bejel and yaws are limited to particular geographical regions of the world: arid, subtropical and humid, tropical areas, respectively [1,4-5,8]. Therefore, both bejel and yaws can be excluded in the differential diagnosis of **KD271**. Acquired syphilis tends to affect the bones of the skull vault and of the rhinomaxillary region of the face in its tertiary stage [1,4-5]. In the latter area, the nasal bones (not observable in our case), the bony nasal septum, the maxillary palatine process, the nasal conchae, and the lateral walls of the maxillary sinuses are the most frequently involved sites, whereas the anterior nasal spine and the maxillary alveolar process are usually spared [4-5,9-10]. Although acquired syphilis cannot be completely rejected as a diagnostic option in **KD271**, as the earliest identified cases of the disease from the present-day territory of Hungary derive from the end of the 15th century CE [11], it is unlikely that acquired syphilis resulted in the development of the bony changes observed in the rhinomaxillary region of the face of **KD271**. Tuberculosis, caused by members of the *Mycobacterium tuberculosis* complex, is primarily a pulmonary disease; nevertheless, it can affect any part of the human body, including the skin [1-2,4,12]. Tuberculosis of the facial skin and soft tissues, facial *lupus vulgaris*, is a rare extra-pulmonary manifestation of the disease [12-14]. Although long-standing facial *lupus vulgaris* can result in secondary involvement of the underlying bone, the maxillary alveolar process is rarely affected by tuberculosis [2,10]. Actinomycosis, caused by *Actinomyces* spp., is a rare medical condition, and actinomycotic involvement of the skeleton is even more uncommon [1,4-5,15-16]. In the skull, the mandible (unchanged in our case) presents the most frequently affected site, whereas the maxilla is an extremely rare localisation of actinomycosis [1,4-5,16]. Based on their localisation preference and rarity, facial *lupus vulgaris* and actinomycosis seem to be less likely to be responsible for the formation of the bony changes detected in the rhinomaxillary region of the face of **KD271**.

Aspergillosis due to *Aspergillus* spp. is a sporadic systemic fungal infection with worldwide distribution that can occasionally affect the skeleton [1,17-19]. If the skull is involved in aspergillosis, the nasal cavity, and the paranasal sinuses and their walls are primarily affected with eventual extension of the infection into the orbits (not changed in our case) [1,17,19]. Similar to aspergillosis, mucormycosis is a rare systemic infection with worldwide distribution that is caused by fungi belonging to the *Mucoraceae* family of the *Mucorales* order [1,17]. In the most common form of the disease, rhinocerebral mucormycosis, the infection can extend into the bones [1,17,19-21]. It spreads in the nasal cavity and paranasal sinuses with subsequent development of sinusitis; from here, the disease frequently progresses into the orbits (not in our case) [1,17,19-21]. In mucormycosis, usually only one of the maxillary sinuses is affected with consequent perforation of the corresponding maxillary palatine process [1,17,19]. Based on the above, aspergillosis and mucormycosis can be excluded in the differential diagnosis of **KD271**.

Sarcoidosis is an uncommon systemic granulomatous disease of unknown aetiology that generally occurs in adults between 25 and 40 years of age and appears to have a predilection for individuals of African descent [17,19,22-24]. Sarcoidosis rarely (~1%) affects the nasal mucosa and consequently the bones of the rhinomaxillary region of the face [12,17,25]. When it does, the nasal bones (not observable in our case) are the primary site of involvement, whereas the anterior nasal spine and the maxilla are not typical localisations of the disease [2,19]. Considering the racial predilection, age and localisation preference, and rarity of sarcoidosis, this medical condition can be ruled out with high certainty as a diagnostic option in **KD271**.

REFERENCES

1) Aufderheide AC, Rodríguez-Martín C. The Cambridge encyclopedia of human paleopathology. Cambridge, UK: Cambridge University Press; 1998.
2) Ortner DJ. Infectious diseases: Tuberculosis and leprosy. In: Ortner DJ, editor. Identification of pathological conditions in human skeletal remains. San Diego, CA, USA: Academic Press; 2003. pp. 227-271.
3) Spigelman M, Rubini M. Paleomicrobiology of leprosy. In: Drancourt M, Raoult DA, editors. Paleomicrobiology of humans. Washington, DC, USA: ASM Press; 2016. pp. 131-142. doi: 10.1128/9781555819170
4) Roberts CA, Buikstra JE. Bakterial infections. In: Buikstra JE, editor. Ortner's Identification of pathological conditions in human skeletal remains. San Diego, CA, USA: Academic Press; 2019. pp. 321-439. doi: 10.1016/B978-0-12-809738-0.00011-9

5) Ortner DJ. Infectious diseases: Treponematosis and other bacterial diseases. In: Ortner DJ, editor. Identification of pathological conditions in human skeletal remains. San Diego, CA, USA: Academic Press; 2003. pp. 273-323.
6) Harper KN, Ocampo PS, Steiner BM, George RW, Silverman MS, Bolotin S, et al. On the origin of the treponematoses: A phylogenetic approach. PLoS Negl Trop Dis. 2008;2(1): e148. doi: 10.1371/journal.pntd.0000148
7) Giacani L, Lukehari SA. The endemic treponematoses. Clin Microbiol Rev. 2014;27(1): 89-115. doi: 10.1128/CMR.00070-13
8) Mediannikov O, Fenollar F, Davoust B, Amanzougaghene N, Lepidi H, Arzouni J-P, et al. Epidemic of venereal treponematosis in wild monkeys: A paradigm for syphilis origin. New Microbes New Infect. 2020;35: 100670. doi: 10.1016/j.nmni.2020.100670
9) Henneberg M, Holloway-Kew K, Lucas T. Human major infections: Tuberculosis, treponematoses, leprosy – A paleopathological perspective of their evolution. PLoS ONE 2021;16(2): e0243687. doi: 10.1371/journal.pone.0243687
10) Møller-Christensen V. Evidence of leprosy in earlier peoples. In: Brothwell DR, Sandison AT, editors. Disease in antiquity. A survey of diseases, injuries and surgery of early populations. Springfield, IL, USA: Charles C. Thomas Publisher; 1967. pp. 295-306.
11) Ősz B, Hajnal K, Marcsik A, Fogas O, Horváth F, Zádori P, et al. Preliminary report on the paleopathological research of the skeletal material from the Szeged medieval castle excavation. Acta Biol Szeged. 2009;53(2): 125-138.
12) Olteanu M, Popescu MR, Nitu M, Calarasu C, Maceseanu AV, Olteanu M. Rare case of pulmonary tuberculosis with hematogenous spread to larynx and skin. Curr Health Sci J. 2016;42(2): 213-216. doi: 10.12865/CHSJ.42.02.15
13) Scollard DM, Dacso MM, Abad-Venida ML. Tuberculosis and leprosy: Classical granulomatous diseases in the twenty-first century. Dermatol Clin. 2015;33(3): 541-562. doi: 10.1016/j.det.2015.03.016
14) Khadka P, Koirala S, Thapaliya J. Cutaneous tuberculosis: Clinicopathologic arrays and diagnostic challenges. Dermatol Res Pract. 2018;2018: 7201973. doi: 10.1155/2018/7201973
15) Valour F, Sénéchal A, Dupieux C, Karsenty J, Lustig S, Breton P, et al. Actinomycosis: Etiology, clinical features, diagnosis, treatment, and management. Infect Drug Resist. 2014;7: 183-197. doi: 10.2147/IDR.S39601

16) Sezer B, Akdeniz BG, Günbay S, Hilmioğlu-Polat S, Başdemir G. Actinomycosis osteomyelitis of the jaws: Report of four cases and review of the literature. J Dent Sci. 2017; 12(3): 301-307. doi: 10.1016/j.jds.2013.02.031
17) Ortner DJ. Infectious diseases: Mycotic, viral, and multicelled parasitic diseases of the human skeleton. In: Ortner DJ, editor. Identification of pathological conditions in human skeletal remains. San Diego, CA, USA: Academic Press; 2003. pp. 325-341.
18) Koehler P, Tacke D, Cornely OA. Aspergillosis of bones and joints – A review from 2002 until today. Mycoses 2014;57(6): 323-335. doi: 0.1111/myc.12165
19) Grauer AL, Roberts CA. Fungal, viral, multicelled parasitic, and protozoan infections. In: Buikstra JE, editor. Ortner's Identification of pathological conditions in human skeletal remains. San Diego, CA, USA: Academic Press; 2019. pp. 441-478. doi: 10.1016/B978-0-12-809738-0.00011-9
20) Garlapati K, Chavva S, Vaddeswarupu RM, Surampudi J. Fulminant mucormycosis involving paranasal sinuses: A rare case report. Case Rep Dent. 2014;2014: 465919. doi: 10.1155/2014/465919
21) Ferguson BJ. Mucormycosis of the nose and paranasal sinuses. Otolaryngol Clin North Am. 2000;33(2): 349-365. doi: 10.1016/s0030-6665(00)80010-9.
22) McCaffrey TV, McDonald TJ. Sarcoidosis of the nose and paranasal sinuses. Laryngoscope 1983;93(10): 1281-1284. doi: 10.1002/lary.1983.93.10.1281
23) Jamilloux Y, Bonnefoy M, Valeyre D, Varron L, Broussolle C, Sève P. Elderly-onset sarcoidosis: Prevalence, clinical course, and treatment. Drugs Aging 2013;30(12): 969-978. doi: 10.1007/s40266-013-0125-5
24) Mirsaeidi M, Machado RF, Schraufnagel D, Sweiss NJ, Baughman RP. Racial difference in sarcoidosis mortality in the United States. Chest 2015;147(2): 438-449. doi: 10.1378/chest.14-1120
25) Kirsten A-M, Watz H, Kirsten D. Sarcoidosis with involvement of the paranasal sinuses – A retrospective analysis of 12 biopsy-proven cases. BMC Pulm Med. 2013;13: 59. doi: 10.1186/1471-2466-13-59
