# Supplementary material for: The two extremes of Hansen’s disease—Different manifestations of leprosy and their biological consequences in an Avar Age (late 7th century CE) osteoarchaeological series of the Duna-Tisza Interfluve (Kiskundorozsma–Daruhalom-dűlő II, Hungary)
Source: PLoS One. 2022 Jun 23;17(6):e0265416. doi: 10.1371/journal.pone.0265416 (PMC9223331; doi:10.1371/journal.pone.0265416)
Supplement: S4 Text — (PDF) [file pone.0265416.s004.pdf]

S4 Text: Non-specific bony changes indicative of motor peripheral neuropathy in leprosy.

Leprous dysfunction of the motor peripheral nerves innervating the hands and feet occurs in more advanced stages of leprosy [1-2]. It is generally preceded and thereby accompanied by sensory nerve impairment [1-3]. The loss of motor function in the peripheral nerves leads to progressive paralysis of individual muscles and muscle groups with secondary deformation and disfigurement of the affected limb(s) [1,4-6].

Paralysis of the flexor and extensor muscles of the hands or feet gives rise to hyperflexion of the interphalangeal joints and hyperextension of the metacarpophalangeal or metatarsophalangeal joints [4,6]. Subsequently, 'claw-hand' or 'claw-toe' deformity can develop [4,6]. In claw-hand deformity consequent to neuropathy of the motor component of the ulnar nerve, the sustained hyperflexion is generally most marked at the proximal interphalangeal joints [4]. The long-standing pressure exerted by the palmar edge of the middle phalangeal base induces bone atrophy at the distal end of the adjoining proximal phalanx [4]. Subsequently, a shallow, occasionally sharply defined groove forms across the palmar surface of the affected proximal phalanx (in the juxta-articular area at its distal end) [4,7]. The palmar edge of the middle phalangeal base can become broad and flat (a smooth remodelled bevelling can be present on it) [4,7]. In the absence of lateral deviation at the proximal interphalangeal joint, the groove extends across the entire width of the palmar surface, whereas lateral deviation at the proximal interphalangeal joint results in unequal grooving [4,7]. In claw-toe deformity consequent to neuropathy of the motor component of the posterior tibial nerve, similar alterations can form on the plantar surface of the foot phalanges [6,8-9].

The loss of motor function in the posterior tibial nerve leads to paralysis of the muscles that are responsible for the maintenance of the longitudinal arch integrity in the foot [7,10]. This results in collapse of the longitudinal arch with secondary development of 'flat-foot' deformity, also known as '*pes planus*' [7,10]. In flat-foot deformity, due to the changed and abnormal mechanical stress between the tarsal bones, dynamic, progressive, plantar displacement of the navicular bone ensues that in turn imposes tensile stress on the dorsal ligaments of the talonavicular, cuneonavicular, and cuboideonavicular joints [7,10]. This chronic ligamentous stress stimulates the formation of exostoses at the attachment sites of the affected dorsal tarsal ligaments [7,10]. These exostoses appear as irregular, smooth ridges of new bone that extend transversely across the dorsal surface of the tarsus – they are most pronounced on the talus and navicular bone [7,10].

REFERENCES

1) Kumar V. Emerging concept on peripheral nerve damage in leprosy. Int J Res Stud Med Health Sci. 2017;2(7): 8-18.
2) Vijayan J, Wilder-Smith EP. Neurological manifestations of leprosy. In: Scollard DM, Gillis TP, editors. International textbook of leprosy. 2018. Available from: https://internationaltextbookofleprosy.org/
3) Ranney DA. The hand in leprosy. The Hand 1973;5(1): 1-9. doi: 10.1016/0072-968x(73)90002-8
4) Andersen JG, Manchester K. Grooving of the proximal phalanx in leprosy: A palaeopathological and radiological study. J Archaeol Sci. 1987;14(1): 77-82. doi: 10.1016/S0305-4403(87)80007-9
5) Amole IO, Adesina SA, Durodola AO, Adeniran A, Awotunde OT, Eyesan SU. Reconstructive surgical correction of ulnar nerve paralytic claw fingers in Hansen's disease patients by lasso procedure. J Case Rep Images Med. 2016;2(1): 31-35. doi: 10.5348/Z09-2016-15-CS-8
6) Roberts CA, Buikstra JE. Bakterial infections. In: Buikstra JE, editor. Ortner's Identification of pathological conditions in human skeletal remains. San Diego, CA, USA: Academic Press; 2019. pp. 321-439. doi: 10.1016/B978-0-12-809738-0.00011-9
7) Crane-Kramer GMM. The paleoepidemiological examination of treponemal infection and leprosy in medieval populations from northern Europe. PhD thesis, University of Calgary (Calgary, AB, Canada). 2000. doi: 10.11575/PRISM/12209
8) Schultz M, Roberts CA. Diagnosis of leprosy in skeletons from an English later medieval hospital using histological analysis. In: Roberts CA, Lewis ME, Manchester K, editors. The past and present of leprosy: Archaeological, historical, palaeopathological and clinical approaches. Oxford, UK: Archaeopress; 2002. pp. 89-104.
9) Lewis M. Infectious diseases II: Infections of specific origin. In: Paleopathology of children. Identification of pathological conditions in the human skeletal remains of non-adults. London, UK: Academic Press; 2018. pp. 151-192. doi: 10.1016/B978-0-12-410402-0.00007-2
10) Andersen JG, Manchester K. Dorsal tarsal exostoses in leprosy: A palaeopathological and radiological study. J Archaeol Sci. 1988;15(1): 51-56. doi: 10.1016/0305-4403(88)90018-0
