# Supplementary material for: The two extremes of Hansen’s disease—Different manifestations of leprosy and their biological consequences in an Avar Age (late 7th century CE) osteoarchaeological series of the Duna-Tisza Interfluve (Kiskundorozsma–Daruhalom-dűlő II, Hungary)
Source: PLoS One. 2022 Jun 23;17(6):e0265416. doi: 10.1371/journal.pone.0265416 (PMC9223331; doi:10.1371/journal.pone.0265416)
Supplement: S5 Text — (PDF) [file pone.0265416.s005.pdf]

S5 Text: Non-specific bony changes indicative of sensory peripheral neuropathy in leprosy.

Leprous dysfunction of the sensory peripheral nerves innervating the hands and feet leads to diminution or complete loss of one or more sensation modalities (e.g., temperature, pain or touch) [1-2]. Generally, the most superficial and thereby the coolest distal terminal branches of the peripheral nerves are the initial sites of involvement in Hansen's disease [3-5]. Leprous neuropathy of these dermal sensory nerve fibres results in gradual and progressive loss of the cutaneous sensation in circumscribed areas with subsequent development of anaesthetic skin patches [3,5]. The earliest sensation modality lost is temperature, followed by pain and light touch [3,5]. Eventually all sensation modalities can become affected [2]. Cutaneous sensory impairment occurs early in the course of leprosy and predisposes patients to unintentional minor superficial trauma of the involved skin area(s) (e.g., burns or cuts) [2-3,6-7]. These repeated unperceived traumata to the soft tissues of the hands and feet result in aseptic inflammation [2]. The inflamed area undergoes aseptic tissue necrosis subsequent to local vascular damage and haemorrhage [2]. Following the development of chronic superficial insensitive ulceration, secondary pyogenic sepsis can ensue [2,6]. This is because the aseptic necrotic area, the aseptic ulcer, provides a portal of entry for invasion by environmental pyogenic bacteria (e.g., *Streptococcus* and *Staphylococcus* spp.) [2,6].

After the loss of cutaneous sensation, deep tissue sensation is often preserved for a long time [2]. Nevertheless, in more advanced stages of Hansen's disease, the gradual, disto-proximal progression of the pathological process along the cutaneous sensory nerve fibres to the larger parent nerve trunk(s) leads to regional sensory and motor dysfunction as these nerve trunks contain not only sensory but also motor nerve fibres [4,8]. The consequent deep tissue anaesthesia and muscle paralysis are generally confined to the innervated territory of the affected peripheral nerve(s) [3,8]. With the loss of deep tissue sensation, the pyogenic sepsis extends from the superficial tissues to the deep soft tissues, bones, and joint cavities [1-2,6-7]. This gives rise to pyogenic septic periostitis, osteitis, osteomyelitis, and/or arthritis of the small bones and joints of the hands and feet with consequent formation of pyogenic septic bony changes, such as surface pitting, subperiosteal new bone formations, cortical bone erosion, lytic or cystic bone lesions, partial or complete bone absorption, subluxation, dislocation, and bony ankylosis [1-2,7]. The nature of these skeletal lesions is similar to that in non-leprous conditions [2,7].

The site of trauma and ulceration is influenced by limb deformities that are consequent to leprous involvement and subsequent impairment of the peripheral motor nerves innervating the hands and feet [2,9]. If there is no significant peripheral motor neuropathy; and therefore, the normal anatomical architecture of the insensitive hand is maintained, trauma and ulceration tend to occur on the palmar surface of the hand, particularly on the ulnar side and at the tips of the fingers [2]. In the presence of claw-hand deformity, that protects the tips of the fingers, the dorsal side of the flexed interphalangeal joints is the most commonly affected area by trauma and ulceration [2]. In the insensitive foot, if the normal anatomical architecture is intact, trauma and ulceration generally occur on the plantar surface, mainly beneath the posteroinferior aspect of the calcaneus and the 1st and 5th metatarsophalangeal joints [2,10]. In the presence of claw-toe deformity, the distal end of the toes can also become involved [2]. If the transverse arch of the insensitive foot is collapsed, there is a tendency for plantar trauma and ulceration to occur beneath the posteroinferior aspect of the calcaneus and the 2nd, 3rd, and 4th metatarsophalangeal joints [2,10]. If the longitudinal arch is lost, particularly with the development of flat-foot deformity, plantar trauma and ulceration tend to affect the mid-foot (in the region of the tarsometatarsal joints, mainly on the fibular side) [2,10].

In the hands, the pyogenic septic bony changes do not usually proceed beyond the metacarpophalangeal joints [2,9]. It is suggested that trauma to the hand would more likely to be noticed by the individual; and therefore, hands and arms would more likely to be protected from further ulceration and secondary pyogenic infection [6]. In the feet, all bones can be affected, and the pyogenic infection can ascend to the long tubular bones of the lower legs [2,6,9]. Different pathways of how these bones become involved were proposed:

1) Pyogenic bacteria from the ulcerated lesions on the plantar surface of the foot spread via the muscle planes or through haematogenous dissemination to the periosteum of the lower leg bones with its subsequent inflammation [2,6];

2) Tibial and/or fibular periostitis is a result of direct extension of the pyogenic infection from an overlying skin ulcer in the lower leg(s) (it is important to note that the skin is in very close contact with the periosteum at the anterior and medial surfaces of the tibia) [6,11]; or

3) Inflammation of the periosteum of the lower leg bones is a biological reaction to the toxins of pyogenic bacteria [2,12].

Periostitis is manifest as surface pitting and marked, longitudinally striated subperiosteal new bone formations that mainly occur in the distal half or two-thirds of the tibial

and/or fibular shafts, without proximal extension to the knee [2,6,9,13-14]. Involvement of the tibia and fibula is often bilateral and symmetrical, and the bony changes are generally most pronounced on the adjacent surfaces of the two lower leg bones [7,13]. In addition, the crural interosseous membrane (the ligamentous structure that connects the corresponding fibula and tibia throughout the length of their shafts) can become involved by the pyogenic infection [6]. This results in its inflammation and subsequent ossification with formation of exostoses at its attachment sites on the lower leg bones [6].

REFERENCES

1) Andersen JG, Manchester K. Grooving of the proximal phalanx in leprosy: A palaeopathological and radiological study. J Archaeol Sci. 1987;14(1): 77-82. doi: 10.1016/S0305-4403(87)80007-9
2) Andersen JG, Manchester K, Roberts CA. Septic bone changes in leprosy: A clinical, radiological and palaeopathological review. Int J Osteoarchaeol. 1994;4(1): 21-30. doi: 10.1002/oa.1390040105
3) Haroun OMOH. Neuropathic pain in leprosy: Deep profiling and stratification of patient groups. PhD thesis, London School of Hygiene and Tropical Medicine (London, UK). 2015. doi: 10.17037/PUBS.02030956
4) Ebenezer GJ, Polydefkis M, Scollard DM. Mechanisms of nerve injury in leprosy. In: Scollard DM, Gillis TP, editors. International textbook of leprosy. 2018. Available from: https://internationaltextbookofleprosy.org/
5) Vijayan J, Wilder-Smith EP. Neurological manifestations of leprosy. In: Scollard DM, Gillis TP, editors. International textbook of leprosy. 2018. Available from: https://internationaltextbookofleprosy.org/
6) Lewis ME, Roberts CA, Manchester K. Inflammatory bony changes in leprous skeletons from the medieval hospital of St. James and St. Mary Magdalene, Chichester, England. Int J Lepr. 1995;63(1): 77-85.
7) Aufderheide AC, Rodríguez-Martín C. The Cambridge encyclopedia of human paleopathology. Cambridge, UK: Cambridge University Press; 1998.
8) Kumar V. Emerging concept on peripheral nerve damage in leprosy. Int J Res Stud Med Health Sci. 2017;2(7): 8-18.
9) Roberts CA, Buikstra JE. Bakterial infections. In: Buikstra JE, editor. Ortner’s Identification of pathological conditions in human skeletal remains. San Diego, CA, USA: Academic Press; 2019. pp. 321-439. doi: 10.1016/B978-0-12-809738-0.00011-9

10) Andersen JG, Manchester K. Dorsal tarsal exostoses in leprosy: A palaeopathological and radiological study. J Archaeol Sci. 1988;15(1): 51-56. doi: 10.1016/0305-4403(88)90018-0
11) Boel LWT, Ortner DJ. Skeletal manifestations of skin ulcer in the lower leg. Int J Osteoarchaeol. 2013;23(3): 303-309. doi: 10.1002/oa.1248
12) Lewis M. Infectious diseases II: Infections of specific origin. In: Paleopathology of children. Identification of pathological conditions in the human skeletal remains of non-adults. London, UK: Academic Press; 2018. pp. 151-192. doi: 10.1016/B978-0-12-410402-0.00007-2
13) Crane-Kramer GMM. The paleoepidemiological examination of treponemal infection and leprosy in medieval populations from northern Europe. PhD thesis, University of Calgary (Calgary, AB, Canada). 2000. doi: 10.11575/PRISM/12209
14) Schultz M, Roberts CA. Diagnosis of leprosy in skeletons from an English later medieval hospital using histological analysis. In: Roberts CA, Lewis ME, Manchester K, editors. The past and present of leprosy: Archaeological, historical, palaeopathological and clinical approaches. Oxford, UK: Archaeopress; 2002. pp. 89-104.
