# Supplementary material for: The two extremes of Hansen’s disease—Different manifestations of leprosy and their biological consequences in an Avar Age (late 7th century CE) osteoarchaeological series of the Duna-Tisza Interfluve (Kiskundorozsma–Daruhalom-dűlő II, Hungary)
Source: PLoS One. 2022 Jun 23;17(6):e0265416. doi: 10.1371/journal.pone.0265416 (PMC9223331; doi:10.1371/journal.pone.0265416)
Supplement: S6 Text — (PDF) [file pone.0265416.s006.pdf]

S6 Text: Non-specific bony changes indicative of autonomic peripheral neuropathy in leprosy.

Leprous dysfunction of the autonomic peripheral nerves innervating the hands and feet is accompanied by circulatory disturbances and consequent changes in the blood oxygen tension of the affected area(s) [1-3]. This, by selectively stimulating regional extracortical osteoclastic and endosteal osteoblastic activity, gives rise to slow, progressive, concentric diaphyseal atrophy/remodelling of the tubular bones of the hands and feet [1,3-4].

Concentric diaphyseal atrophy is characterised by bone absorption on the outer cortical surface with concurrent bone deposition on the inner cortical surface in all diameters of the affected bone [1-4]. It leads to gradual and progressive diminution of the overall diameter of the diaphysis and medullary cavity, especially at the diaphyseal site where the pathological process commences and is maximal in progress – at the mid-shaft in the phalanges and at the distal third of the shaft in the metacarpals and metatarsals [1-4]. Thus, in early stages of the concentric diaphyseal atrophy, the affected bone has an hourglass shape [5]. Eventually, the pathological process results in a diaphyseal site that consists of only compact bone, with complete obliteration of the medullary cavity (the thickness of the cortical bone is maintained or even increased) [1-2]. As the bone absorption on the outer cortical surface continues, it can lead to further thinning and consequent pathological fracture of the weakened diaphyseal site as it is no longer able to withstand biomechanical stress [1-3]. The fracture results in a proximal and distal fragment of the affected bone with disunion between the two [1-2]. Later in the pathogenesis, the distal fragment usually completely absorbs due to coincident acro-osteolysis (loss of bone via diffuse absorption), while the proximal fragment continues to remodel and, as it gradually thins, can become pointed at its distal end ("pencilling" or "shark tooth" deformity) [1-4]. Ultimately, the proximal fragment can also absorb as a result of simultaneous concentric diaphyseal atrophy and acro-osteolysis [1-4]. In the metatarsals, a solely mediolateral, 'knife-edge' remodelling can also ensue, where the bone absorption on the outer cortical surface with concomitant bone deposition on the inner cortical surface is most marked at the medial and lateral sides of the diaphysis (the superoinferior diameter of the affected bone is maintained) [1,3]. This results in a knife-shaped appearance of the affected bone with sharp inferior and superior borders [1,3].

In the hands, the bone resorption begins at the tip of one or more distal phalanges and continues to progress proximally [2,4]. It can extend to the middle or later to the proximal phalanges, or occasionally even to the metacarpals [2,4]. In the feet, the concentric diaphyseal

atrophy commences at the metatarsophalangeal joints (in the metatarsals and proximal phalanges), and often spares the distal phalanges [2-4]. The above-mentioned resorptive changes can terminate in subluxation or dislocation of the affected bones of the hands and/or feet [2-3,6]. Subluxation of the phalanges at the interphalangeal, metacarpophalangeal or metatarsophalangeal joints and subsequent formation of circumferential periarticular osteophytes, that develop in response to traction on the joint capsule, produce a cup deformity at the proximal end of the phalanges [2-3,6].

REFERENCES

1) Andersen JG, Manchester K, Ali RS. Diaphyseal remodelling in leprosy: A radiological and palaeopathological study. Int J Osteoarchaeol. 1992;2(3): 211-219. doi: 10.1002/oa.1390020305
2) Ortner DJ. Infectious diseases: Tuberculosis and leprosy. In: Ortner DJ, editor. Identification of pathological conditions in human skeletal remains. San Diego, CA, USA: Academic Press; 2003. pp. 227-271.
3) Roberts CA, Buikstra JE. Bakterial infections. In: Buikstra JE, editor. Ortner’s Identification of pathological conditions in human skeletal remains. San Diego, CA, USA: Academic Press; 2019. pp. 321-439. doi: 10.1016/B978-0-12-809738-0.00011-9
4) Aufderheide AC, Rodríguez-Martín C. The Cambridge encyclopedia of human paleopathology. Cambridge, UK: Cambridge University Press; 1998.
5) Crane-Kramer GMM. The paleoepidemiological examination of treponemal infection and leprosy in medieval populations from northern Europe. PhD thesis, University of Calgary (Calgary, AB, Canada). 2000. doi: 10.11575/PRISM/12209
6) Andersen JG, Manchester K, Roberts CA. Septic bone changes in leprosy: A clinical, radiological and palaeopathological review. Int J Osteoarchaeol. 1994;4(1): 21-30. doi: 10.1002/oa.1390040105
