# Supplementary material for: The two extremes of Hansen’s disease—Different manifestations of leprosy and their biological consequences in an Avar Age (late 7th century CE) osteoarchaeological series of the Duna-Tisza Interfluve (Kiskundorozsma–Daruhalom-dűlő II, Hungary)
Source: PLoS One. 2022 Jun 23;17(6):e0265416. doi: 10.1371/journal.pone.0265416 (PMC9223331; doi:10.1371/journal.pone.0265416)
Supplement: S7 Text — (PDF) [file pone.0265416.s007.pdf]

S7 Text: Differential diagnoses of the postcranial bony changes indicative of leprous autonomic peripheral neuropathy that were observed in KD520.

The most relevant differential diagnoses of the postcranial skeletal lesions indicative of leprous autonomic peripheral neuropathy that were observed in **KD520** are certain types of erosive arthropathies (e.g., rheumatoid arthritis and psoriatic arthritis), hereditary sensory and autonomic neuropathies, and *diabetes mellitus* [1].

Rheumatoid arthritis is a chronic, systemic joint disease of autoimmune in nature that primarily targets the synovial lining [2-6]. It is characterised by bilaterally symmetrical involvement of the peripheral synovial joints – usually multiple sites are concurrently affected by the disease [2,5,7-8]. Rheumatoid arthritis can occur at any age but generally, it arises in the fourth or fifth decade of life [3,5,7-8]. Females are more susceptible for developing rheumatoid arthritis than males, with a ratio of about 3:1 between the two sexes [4,7-8]. Any synovial joint can become involved in rheumatoid arthritis but it initially commences in the small joints of the hands and feet, especially in the metacarpophalangeal and proximal interphalangeal joints of the hands [2,4-8]. The feet tend to be less frequently affected by the disease [2,7-8]. Later, rheumatoid arthritis can progress to the larger joints of the limbs, such as the elbow, shoulder, ankle or knee joint [2,4-8]. Similar to rheumatoid arthritis, psoriatic arthritis is a chronic systemic autoimmune disease that involves the synovial joints [2,8,10]. It occurs in individuals with cutaneous psoriasis – up to 30% of the patients will develop psoriatic arthritis [2,8,10-12]. In contrast to rheumatoid arthritis, asymmetrical and oligoarticular joint involvement is not uncommon in psoriatic arthritis [2,8-10]. Females and males are almost equally affected by psoriatic arthritis, with the average age at disease onset being around the fourth to fifth decade of life [8,11-12]. Similar to rheumatoid arthritis, the small synovial joints of the hands and feet, especially the distal interphalangeal joints, are the most commonly affected sites in psoriatic arthritis [2,8-10]. The hands tend to be more frequently involved than the feet [9]. Later in the pathogenesis, the larger synovial joints of the limbs, as well as the synovial joints of the axial skeleton can also become affected by psoriatic arthritis [2,8-11]. Rheumatoid arthritis and psoriatic arthritis primarily result in joint changes, such as synovial inflammation, articular cartilage and subchondral bone erosion, subluxation, and bony ankylosis [2,6-10]. In advanced stages of these diseases, the erosive lesions can progress to development of a 'pencil-in-cup' deformity of the involved hand and/or foot joints [2,6-10]. Based on the localisation, age and/or sex preference of rheumatoid arthritis and psoriatic arthritis, they could be potential diagnostic options in **KD520**. However, the macromorphological appearance of the detected metatarsal

and phalangeal alterations (the absence of joint changes, such as bone erosion in the juxta-articular area) is not consistent with the pencil-in-cup deformity that can be observed in cases with rheumatoid arthritis or psoriatic arthritis. Therefore, these medical conditions can be excluded in the differential diagnosis of **KD520**.

Hereditary sensory and autonomic neuropathies are a heterogeneous group of very rare, genetically determined disorders of the peripheral nervous system [13-15]. They are associated with sensory, autonomic and/or motor dysfunction of variable predominance due to different genetic errors that affect specific aspects of the development of small nerve fibres [13-15]. Hereditary sensory and autonomic neuropathies are currently classified into five types (HSAN1, HSAN2, HSAN3, HSAN4, and HSAN5) based on inheritance pattern, age at onset, and clinical features [14,16]. The autosomal recessive types (HSAN2–5) usually have a very early, often congenital onset [13,15]. In contrast to these forms, the only autosomal dominant type (HSAN1 or Thévenard's disease) generally presents in the second or third decade of life (or even later) [13-15,17]. Among other bony changes, concentric diaphyseal atrophy of the foot bones has also been recorded in patients with different types of hereditary sensory and autonomic neuropathies [13]. Nevertheless, based on the severity and extent of the observed alterations, the early-onset types of hereditary sensory and autonomic neuropathies can be ruled out with high certainty in the differential diagnosis of **KD520**. Although Thévenard's disease cannot be completely rejected as a diagnostic option in **KD520**, considering its rarity, it is unlikely that this medical condition was responsible for the development of the concentric diaphyseal atrophy of the middle-aged female's foot bones.

Diabetes mellitus is a metabolic disease with its most frequent forms being 1) the childhood-onset type 1 *diabetes mellitus* that is associated with failure in insulin production; and 2) the adult-onset type 2 *diabetes mellitus* that is characterised by insulin resistance and reduction of insulin production [18]. Diabetic peripheral neuropathy is a common complication of *diabetes mellitus* that can develop in all forms of the disease [19-21]. It occurs in up to 50% of patients with *diabetes mellitus* and usually presents as symmetrical distal polyneuropathy that accounts for about 75% of the cases [19-21]. In long-standing diabetic conditions, sensory and autonomic impairment, and later to a lesser extent, motor dysfunction in the peripheral nerves can lead to the development of bony changes (e.g., concentric diaphyseal atrophy of the foot bones) and associated limb deformation [2,20,22]. Considering the age at death of **KD520**, adult-onset type 2 *diabetes mellitus* could be a potential diagnostic option in the middle-aged female. However, based on the absence of bony and dental changes indicative of *diabetes mellitus* in other areas of the skeleton (e.g., signs of adhesive capsulitis, diffuse idiopathic

skeletal hyperostosis or tooth pathologies, such as dental caries, periodontitis or *ante-mortem* tooth loss), *diabetes mellitus* seems to be less likely to be responsible for the formation of concentric diaphyseal atrophy of the foot bones of **KD520** [23].

REFERENCES

1) Ortner DJ. Differential diagnosis of skeletal lesions in infectious disease. In: Pinhasi R, Mays S, editors. Advances in human palaeopathology. Chichester, UK: John Wiley & Sons, Ltd.; 2008. pp. 191-214.
2) Aufderheide AC, Rodríguez-Martín C. The Cambridge encyclopedia of human paleopathology. Cambridge, UK: Cambridge University Press; 1998.
3) Guo Q, Wang Y, Xu D, Nossent J, Pavlos NJ, Xu J. Rheumatoid arthritis: Pathological mechanisms and modern pharmacologic therapies. Bone Res. 2018;6: 15. doi: 10.1038/s41413-018-0016-9
4) Smolen JS, Aletaha D, Barton A, Burmester GR, Emery P, Firestein GS, et al. Rheumatoid arthritis. Nat Rev Dis Primers 2018;4: 18001. doi: 10.1038/nrdp.2018.1
5) Bullock J, Rizvi SAA, Saleh AM, Ahmed SS, Do DP, Ansari RA, et al. Rheumatoid arthritis: A brief overview of the treatment. Med Princ Pract. 2019;27(6): 501-507. doi: 10.1159/000493390
6) Boldeanu MV, Ionescu AR, Popoviciu VH, Bărbulescu AL, Dinescu ŞC, Siloşi I, et al. Diagnostic challenges and management update in rheumatoid arthritis. In: Mohammed RHA, editor. Rheumatoid arthritis – Other perspectives towards a better practice. London, UK: IntechOpen; 2020. pp. 294-427. doi: 10.5772/intechopen.91965
7) Ortner DJ, Putschar WGJ. Identification of pathological conditions in human skeletal remains. Washington, DC, USA: Smithsonian Institution Press; 1981. pp. 403-411.
8) Ortner DJ. Erosive arthropathies, enthesopathies, and miscellaneous pathological conditions of joints. In: Ortner DJ, editor. Identification of pathological conditions in human skeletal remains. San Diego, CA, USA: Academic Press; 2003. pp. 561-587.
9) Ory PA, Gladman DD, Mease PJ. Psoriatic arthritis and imaging. Ann Rheum Dis. 2005;64(Suppl. 2): 55-57. doi: 10.1136/ard.2004.033928
10) Merola JF, Espinoza LR, Fleischmann R. Distinguishing rheumatoid arthritis from psoriatic arthritis. RMD Open 2018;4: e000656. doi: 10.1136/rmdopen-2018-000656
11) Caso F, Tasso M, Chimenti MS, Navarini L, Perricone C, Girolimetto N, et al. Late-onset and elderly psoriatic arthritis: Clinical aspects and management. Drugs and Aging 2019;36(10): 909-925. doi: 10.1007/s40266-019-00688-3

12) Ocampo DV, Gladman D. Psoriatic arthritis. F1000Res. 2019;8(F1000 Faculty Rev): 1665. doi: 10.12688/f1000research.19144.1

13) Burg D, Pongratz D, Burg G. Hereditary sensory and autonomic neuropathies – Classification and clinical characteristics. In: Asbury AK, Budka H, Sluga E, editors. Sensory neuropathies. Vienna, Austria: Springer; 1995. pp. 57-73. doi: 10.1007/978-3-7091-6595-9_7

14) Houlden H, King R, Blake J, Groves M, Love S, Woodward C, et al. Clinical, pathological and genetic characterization of hereditary sensory and autonomic neuropathy type I (HSAN I). Brain 2006;129: 411-425. doi: 10.1093/brain/awh712

15) Axelrod FB, Gold-von Simson G. Hereditary sensory and autonomic neuropathies: Types II, III, and IV. Orphanet J Rare Dis. 2007;2: 39. doi: 10.1186/1750-1172-2-39

16) Landrieu P, Baets J, De Jonghe P. Hereditary motor-sensory, motor and sensory neuropathies in childhood. In: Dulac O, Lassonde M, Sarnat HB, editors. Handbook of clinical neurology. Volume 113: Pediatric neurology, Part III. Amsterdam, Netherlands: Elsevier; 2013. pp. 1413-1432. doi: 10.1016/B978-0-444-59565-2.00011-3

17) Nicolić B, Mišović M, Zorić M, Matović D, Aleksić A. Thevenard's disease – A hereditary sensory and autonomic neuropathy type I. Serbian J Dermatol Venereol. 2010;2(2): 59-64. doi: 10.2478/v10249-011-0023-y

18) Tan SY, Wong JLM, Sim YJ, Wong SS, Elhassan SAM, Tan SH, et al. Type 1 and 2 diabetes mellitus: A review on current treatment approach and gene therapy as potential intervention. Diabetes Metab Syndr. 2019;13(1): 364-372. doi: 10.1016/j.dsx.2018.10.008

19) Bansal V, Kalita J, Misra UK. Diabetic neuropathy. Postgrad Med J. 2006;82(965): 95-100. doi: 10.1136/pgmj.2005.036137

20) Feldman EL, Callaghan BC, Pop-Busui R, Zochodne DW, Wright DE, Bennett DL, et al. Diabetic neuropathy. Nat Rev Dis Primers 2019;5(1): 41. doi: 10.1038/s41572-019-0092-1

21) Yang H, Sloan G, Ye Y, Wang S, Duan B, Tesfaye S, et al. New perspective in diabetic neuropathy: From the periphery to the brain, a call for early detection, and precision medicine. Front Endocrinol. 2020;10: 929. doi: 10.3389/fendo.2019.00929

22) Clouse ME, Gramm HF, Legg M, Flood T. Diabetic osteoarthropathy. Clinical and roentgenographic observations in 90 cases. Am J Roentgenol Radium Ther Nucl Med. 1974;121(1): 22-34. doi: 10.2214/ajr.121.1.22

23) Dupras TL, Williams LJ, Willems H, Peeters C. Pathological skeletal remains from ancient Egypt: The earliest case of diabetes mellitus?. Pract Diab Int. 2010;27(8): 358-363a. doi: 10.1002/pdi.1523
