# Supplementary figures and images for: The two extremes of Hansen’s disease—Different manifestations of leprosy and their biological consequences in an Avar Age (late 7th century CE) osteoarchaeological series of the Duna-Tisza Interfluve (Kiskundorozsma–Daruhalom-dűlő II, Hungary)
Source: PLoS One. 2022 Jun 23;17(6):e0265416. doi: 10.1371/journal.pone.0265416 (PMC9223331; doi:10.1371/journal.pone.0265416)

**S1 Figure: Demographic profile of the Avar Age cemetery of Kiskundorozsma–Daruhalom-dűlő II (n=94).**

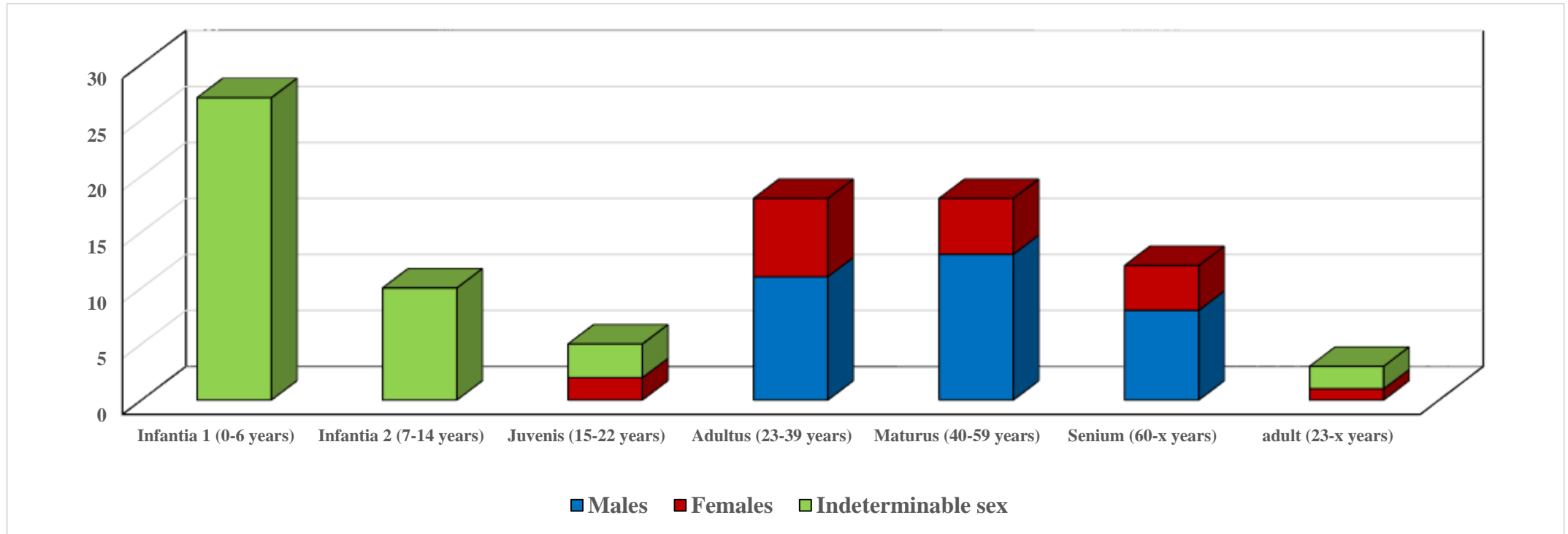

Supplement: S1 Fig — (PDF) [file pone.0265416.s008.pdf]
