# Supplementary material for: The two extremes of Hansen’s disease—Different manifestations of leprosy and their biological consequences in an Avar Age (late 7th century CE) osteoarchaeological series of the Duna-Tisza Interfluve (Kiskundorozsma–Daruhalom-dűlő II, Hungary)
Source: PLoS One. 2022 Jun 23;17(6):e0265416. doi: 10.1371/journal.pone.0265416 (PMC9223331; doi:10.1371/journal.pone.0265416)
Supplement: Attachment — Submitted filename: response_to_reviewers.doc [file pone.0265416.s009.doc]

Dr JJ Cray Jr., PhD

Academic Editor

PLOS ONE

October 19, 2021

Dear Dr JJ Cray Jr.,

I am very thankful for the reviewers’ insightful and constructive comments regarding our manuscript entitled “The two extremes of Hansen’s disease – Different manifestations of leprosy and their biological consequences in an Avar Age (late 7^th^ century CE) osteoarchaeological series of the Duna-Tisza Interfluve (Kiskundorozsma–Daruhalom-dűlő II, Hungary)” that was submitted to the PLOS ONE (manuscript ID: PONE-D-21-21682). I am sure that the reviewers helped us to improve the quality of our manuscript. The main text has been modified following the reviewers’ suggestions. Furthermore, some of the figures were replaced to avoid copyright issues that were mentioned in the editorial letter. The revised files have been uploaded to the submission site of the PLOS ONE.

**Responses to the suggestions:**

1) **Reviewer 1** commented that in the manuscript, we should explain why we use both “Hansen’s disease” and “leprosy” throughout the text. In **Reviewer 1**’s opinion, we should use a single nomenclature for the disease – “Hansen’s disease”, “leprosy”, or “mycobacterial neurodermatosis”. We agree with **Reviewer 1** that “mycobacterial neurodermatosis” is a more neutral term, and considering the stigma and prejudice that is related to leprosy, “mycobacterial neurodermatosis” could be an appropriate choice instead of “leprosy”. However, to the best of our knowledge, in the palaeopathological literature, “leprosy” is the most frequently used nomenclature for the disease. Recently, “Hansen’s disease” started to be in use but unfortunately, we have never seen the term “mycobacterial neurodermatosis” in palaeopathological papers. In most of the articles we could find in the palaeopathological literature, “Hansen’s disease” was mentioned as another name for leprosy but then the term “leprosy” was used throughout the text. In the minority of the palaeopathological papers, both “leprosy” and “Hansen’s disease” were used throughout the text. As, in our opinion, palaeopathologists would represent the main readership of our paper, we would like to keep the term “leprosy”, as this term would be the most familiar for them. If **Reviewer 1** agrees, we would like to use the term “Hansen’s disease”, as well. On the one hand, by using both terms, we would like to avoid word repetition, especially in sentences where the name of the disease would be mentioned twice. On the other hand, by using not only “leprosy” but “Hansen’s disease”, we would like to contribute to this term to become more frequently used in the palaeopathological literature. We hope that by defining “leprosy” and “Hansen’s disease’ as interchangeable names of the same disease in the first sentence of our manuscript, we can avoid confusion in the reader that could derive from the usage of both terms.

2) **Reviewer 1** noted that “the long introductory sections (most of the text/description) for bony changes could fit better in the Introduction” instead of the Discussion. **Reviewer 2** also mentioned that we should consider removing “lines 416-453, 543-574, 625-690, and 771-808” from the Discussion as “these parts are not properly discussion and are not necessary to discuss the two cases”. We agree with the reviewers that the Discussion was not the best choice for these parts to be placed but we did not want to put these long texts into the Introduction because we thought that it would have become too long. On the other hand, we thought that by putting this general information into the Discussion, right before the detailed discussion of the detected bony changes and their biological consequences, it would make it easier for the reader to connect the medical and palaeopathological literature data to the bony changes observed in KD271 and KD520. We also agree with **Reviewer 1** that the aforementioned introductory information is important and useful, especially if the reader is not an expert in the palaeopathological diagnosis of leprosy. Therefore, instead of completely deleting these parts from our manuscript, following the reviewers’ advice, we removed this introductory information from the Discussion and put it into the Supporting Information (S2, S4, S5, and S6 Texts). This way, the Introduction did not become annoyingly long and the introductory general information from the Discussion remained available for the reader. As the replacement of these paragraphs changed the order of some references, they were renumbered accordingly (both in the main text and reference list). Furthermore, following **Reviewer 1**’s suggestion, we kept the subtitles in the Discussion and left “some general osteological information to explain or justify why KD271 could be associated with LL, and KD520 with TL”.

3) **Reviewer 1** commented that we should expand our argumentation why we think that in KD520, TL is the most likely diagnosis, as based on the original version of our argumentation, it seems that the diagnosis of TL for KD520 is weak or not definitive. We agree with **Reviewer 1** that we should expand this aspect in the revised version of our manuscript, as we only briefly mentioned in the Conclusions part of the original version that “Although a number of differential diagnoses need to be considered for the individual bony changes observed in the skeleton of KD271 and KD520, based on the nature, association, and distribution pattern of the registered alterations, they are most consistent with HD in both individuals.”. Even in KD271, where not only the postcranial skeleton but also the rhinomaxillary region seemed to be affected by leprosy, we had to provide a detailed differential diagnosis of the observed rhinomaxillary bony changes (S2 Text in the original version) to exclude the other possible aetiologies (e.g., syphilis or tuberculosis). In the case of KD271, we were lucky that the diagnosis of HD could be established based not only on the observed macromorphological bony changes and their association with each other but on the results of the aDNA analysis, as the sample from the hard palate appeared to be *M. leprae* DNA-positive. We also agree with both reviewers that the diagnosis of TL in osteoarchaeological series is more complex than that of LL – in the palaeopathological literature, there have been only a few attempts to identify TL cases. Based on the above, in the revised version of our manuscript, we highlighted that the diagnosis of HD is weaker in KD520 than in KD271. We agree with **Reviewer 2** that a positive aDNA result could strengthen the diagnosis of leprosy in KD520. However, the chance for successfully detecting *M. leprae* DNA in historic TL cases is very low. On the one hand, TL is a paucibacillary form of Hansen’s disease (in contrast to LL, which is a multibacillary form). On the other hand, the (postcranial) bony changes developing in TL cases, such as KD520, are not secondary to direct invasion of the affected bones by leprosy bacilli but to peripheral neuropathy (in contrast to the rhinomaxillary changes observed in LL cases, such as KD271, that directly result from the presence of the pathogens in the affected bones). Therefore, it is not surprising that the aDNA analysis for *M. leprae* gave negative results in KD520 as very likely, leprosy bacilli have not been present in her bones even when she was alive (reference 100 in the revised manuscript). Furthermore, as an adage in aDNA says: “absence of evidence is not evidence of absence” (reference 101 in the revised manuscript). Thus, even if the aDNA analysis could not strengthen the macromorphological diagnosis of TL in KD520, in our opinion, based on the detailed differential diagnosis (S3 Text in the original version of our manuscript), in which we excluded the other possible aetiologies, the macromorphological appearance and association of the observed bony changes (i.e., concentric diaphyseal atrophy and inflammatory bony changes of the small bones of the feet, and the tibiae and fibulae), as well as the high number of leprosy cases in the Kiskundorozsma–Daruhalom-dűlő II osteoarchaeological series (among which three proved to be *M. leprae* DNA-positive), TL seems to be the most likely diagnosis in KD520. Following **Reviewer 1**’s advice, this aspect was expanded in the revised version of our manuscript. Because of this, two extra references (references 100 and 101) were added to the manuscript, both in the main text and reference list. It seems that our phrasing in the original manuscript has been unclear, as we already mentioned in that version that an aDNA analysis for *M. leprae* was performed not only on KD271 but also on KD520. However, as it could have been expected, it gave positive results only in KD271 (lines 255–259 and 877–878 in the original version of the manuscript). We are really sorry for the misunderstanding generated by our inappropriate phrasing in the original version of the manuscript. Following **Reviewer 2**’s suggestion, to clarify that there was an aDNA analysis for *M. leprae* on KD520 and it gave negative results, the relevant sentence was supplemented in the Conclusions.

4) Following **Reviewer 2**’s suggestion, “type” was replaced by “leprosy” in the Keywords (“lepromatous type” was changed to “lepromatous leprosy” and “tuberculoid type” was changed to “tuberculoid leprosy”).

5) Following **Reviewer 2**’s comment, “Hungary” and the total number of skeletons from the archaeological site of Kiskundorozsma-Daruhalom-dűlő (“n=94”) was added in line 29 in the Abstract.

6) **Reviewer 2** noted that we should consider removing the text between lines 57–73 (in the original version of our manuscript). Following **Reviewer 2**’s suggestion, some of the sentences between lines 57–73 were deleted. Nonetheless, some of the sentences between lines 57–73 were kept, as in our opinion, they contain important information that are later referred in the text or necessary for the reader to understand the following paragraphs in the Introduction, Discussion, and Supporting Information parts of the manuscript (e.g., the main portal of entry and exit for leprosy bacilli is the nasal mucosa; not all the infected individuals develop active disease and consequently, bony changes due to genetic susceptibility; and leprosy has a long incubation period and in some cases, the infection can spontaneously resolve).

7) **Reviewer 2** suggested that we should check the spelling of “achro-osteolysis”. In a number of papers we used as references (e.g., references 11, 20, 52, and 56 in the original version of our manuscript), it was spelled as “achro-osteolysis”. This is why we used this spelling. However, following **Reviewer 2**’s advice, the spelling was changed to “acro-osteolysis” throughout the text.

8) Following **Reviewer 2**’s comment, we replaced “hand and/or foot bones” by “hand and/or foot phalanges, metacarpals, and metatarsals” in line 130 (in the original version of our manuscript).

9) **Reviewer 2** mentioned that we should “add the specific methods used to estimate sex and age at death of skeletons KD271 and KD520”, because “it is unclear how these parameters were estimated for these individuals (lines 247–250)”. We agree with **Reviewer 2** that, as we put all the references of the applied methods to the end of the relevant sentence in lines 247–250 of the original manuscript, we did not specify, which methods were used for age at death estimation and which ones for sex determination. To clarify this, we put the appropriate references after “age at death” and “sex” in the relevant sentence in the revised version: “Age at death [36-45] was estimated and sex [46] was determined applying standard macromorphological methods of biological anthropology.” As the order of the references changed, we renumbered them (both in the main text and reference list).

10) Following **Reviewer 2**’s advice, a diagram presenting the demographic profile of the examined skeletal series was added as Supplementary figure 1.

11) **Reviewer 2** asked us to “add a photo (to be included in Fig. 4) showing the nasal surface of the palatine process of sk. 271” and “add the dimensions of the perforation of the palatine process (line 273)”. Unfortunately, we cannot provide such a photo and cannot add the dimensions of the perforation. As it has been mentioned in the original version of our manuscript, an aDNA analysis was performed on KD271 and the sample was taken from the hard palate; consequently, the majority of the hard palate was destroyed. Therefore, during the macromorphological re-evaluation of KD271, we could not take photos of the nasal and oral surfaces of the maxillary palatine process and could not measure the perforation of the hard palate. To describe the lesions of the nasal and oral surfaces of the hard palate, we used the original, very short description of KD271 that was provided by Marcsik and her co-workers in 2007 (reference 28 in the original version of our manuscript) and the photos that were taken by them back in the 2000s. Unfortunately, in the original description, there was no information regarding the dimensions of the perforation, and among the original photos taken by Marcsik and her colleagues in the 2000s, Figures 4E and 4F were the only ones which we could use in our manuscript to present the oral and nasal surfaces of the hard palate, respectively. This is why we highlighted in the legend of Figure 4 that “Photos A–D were taken after sampling for aDNA analysis, whereas photos E–F were taken before sampling for aDNA analysis.”. Nevertheless, in our opinion, the scale on Figure 4E can help the reader to at least estimate the dimensions of the perforation of the hard palate. Since, based on this scale, it would not be an accurate way to measure the exact dimensions of the lesion, if **Reviewer 2** agrees, we would not include these estimates to the manuscript.

12) Following **Reviewer 2**’s suggestion, “tarsal bones of the feet” was replaced by “tarsal bones” in line 307 (in the original version of our manuscript), “pathognomonic” was removed from line 404, and “of the face” was deleted from line 456 (in the original version of our manuscript).

13) **Reviewer 2** asked us to avoid the term “periostitis” and use “periosteal new bone” instead. Unfortunately, we cannot avoid the term “periostitis”, as in the sentences we used it, we wanted to refer to the condition, i.e., inflammation of the periosteum, that results in deposition of new bone on the extracortical surface of the affected bone subjacent to the inflamed periosteum. In those sentences, where we wanted to refer to the signs of periostitis, i.e., the periosteal new bone depositions, that could have been observed on the skeletal remains of both KD271 and KD520, we used the term “subperiosteal new bone formation” as in those cases, we wanted to refer not to the condition leading to the development of the detected subperiosteal new bone formations but the bony changes themselves. Throughout the manuscript, we tried to be very cautious about when we referred to the condition, i.e., periostitis, and when to its bony signs, i.e., subperiosteal new bone formations. Therefore, if **Reviewer 2** agrees, we would like to use the term “periostitis” instead of “periosteal new bone” in the relevant sentences.

14) **Reviewer 2** asked us to mention “what methods were applied to evaluate osteoporosis” as “it is unclear and needs clarification”. Unfortunately, during the macromorphological re-evaluation of the skeletal remains of KD271 and KD520, they could be examined only with the naked eye (no radiological investigations could be conducted). Therefore, the assessment of the affected limb bones regarding the signs of osteoporosis was very limited. Nevertheless, no evident macromorphological signs of osteoporosis (e.g., substantially reduced cortical thickness, fragility fractures or unusually lightweight bones) could be detected in the affected limb bones of KD271 and KD520 (e.g., references 11, 20, and 52 in the original version of the manuscript). To further highlight this aspect, it would be very useful if a detailed radiological examination could also be performed on the skeletal remains of KD271 and KD520 in the future. Following **Reviewer 2**’s comment, this part of the Discussion was supplemented.

15) Regarding the discussion of the non-specific bony changes that was made by dividing in three parts (motor, sensory, and autonomic peripheral neuropathy), **Reviewer 2** noted that “I’m not sure if this “classic” artificial division, influenced by the radiological literature, is the most useful one.” We chose this division because in our opinion, it is the most appropriate one to present the association between the macromorphological bony changes that were detected with the naked eye in the skeleton of KD271 and KD520 and the exact aetiology, i.e., sensory, motor, and autonomic peripheral neuropathy, that led to the development of the observed alterations. For this reason, if **Reviewer 2** agrees, we would like to keep this division.

16) It was stated in the editorial letter that we should “provide additional information regarding the specimens used in your study. Ensure that you have reported specimen numbers and complete repository information, including museum name and geographic location”. Following this comment, in the Ethics Statement, the specimen numbers (KD271 and KD520) were complemented by the inventory and grave numbers of the evaluated individuals, and the geographic location of the two skeletons (KD271 and KD520) was supplemented by the postal address of the Department of Biological Anthropology, University of Szeged (Szeged, Hungary).

17) In the editorial letter, it was stated that “in order to use the direct billing option the corresponding author must be affiliated with the chosen institute. Please either amend your manuscript to change the affiliation or corresponding author, or email us at plosone@plos.org with a request to remove this option”. We do not really understand why it seems that our corresponding author (Olga Spekker) is not affiliated with the chosen institute (University of Szeged). On the title page of the original version of our manuscript, both the University of Szeged and the Eötvös Loránd University were mentioned as her affiliations, since she has a job at both universities. In the online submission system, she named the University of Szeged as her affiliation, since she applied for funding to the Szeged Open Access Fund to cover the publication fees. Therefore, in our opinion, it is not necessary to change the corresponding author or to change the affiliation of the corresponding author. We hope that after rechecking of the affiliation of the corresponding author (Olga Spekker) in the manuscript file and online submission system, the Journal Office will not find it necessary to amend our manuscript (i.e., to change the corresponding author or the affiliation of the corresponding author).

18) It was stated in the editorial letter that some of the images in our figures may be copyrighted. To avoid copyright issues, Figures 1A, 2C, and 2D were replaced. Unfortunately, as we do not know the original source of the map and the drawing of a skeleton that were used after substantial modifications in Figures 1 and 2 in the original version of our manuscript, we cannot obtain permission from the original copyright holder. For the same reason, we do not know if the above-mentioned source images are actually copyrighted or not. That is why we decided to supply replacement figures in the revised version of our manuscript. It should be mentioned that in Figures 2C and 2D, the left tibia of KD271 and KD520 are missing from the replacement photos, whereas in the original image (drawing of a skeleton), they were marked as available for examination. This is because these bones were available for investigation back in the 2000s (this preliminary evaluation was performed by Antónia Marcsik and Erika Molnár, two of the co-authors of this manuscript) but later, have been transported abroad for further analyses. Unfortunately, the above-mentioned two tibiae have never been returned to the University of Szeged (Szeged, Hungary); therefore, they were not available for re-evaluation and photo-shooting, performed only a couple of months ago. For the same reason, similar to the bony changes of the hard palate of KD271, to describe the lesions of these two tibiae, we used the original descriptions that were provided by Marcsik and her co-workers in 2007 (reference 28 in the original version of our manuscript) and photos that were taken by them back in the 2000s. As they have not taken photos of the complete skeletons of KD271 and KD520 to present their completeness, we had to take new photos, but without the left tibiae, to use them as replacement images for Figures 2C and 2D that present the completeness of the two examined skeletons. As for Figures 3–11, most of the pictures were taken and edited by Luca Kis, one of the co-authors, for this manuscript right before the original submission; they have never been published elsewhere.  Figures 4E and 4F were taken by another co-author, Erika Molnár, back in the 2000s, but they have never been published elsewhere either. Therefore, in our opinion, the aforementioned figures are not copyrighted and we can use them freely in our manuscript without providing a Content Permission Form. We hope that after rechecking our provided figures, the Journal Office will not find it necessary to replace Figures 3–11.

In the revised version of our manuscript, we tried to execute all suggestions of the reviewers. I hope this new version will be suitable for publication in the PLOS ONE.

Thank you again for the reviewers’ insightful and constructive comments and your editorial work!

Yours sincerely,

Dr Olga Spekker, PhD

Postdoctoral researcher

Department of Biological Anthropology

University of Szeged

Közép fasor 52, H-6726 Szeged, Hungary

Email: [olga.spekker@gmail.com](mailto:olga.spekker@gmail.com)

Tel: +36 20 807 72 94
