# Supplementary material for: The two extremes of Hansen’s disease—Different manifestations of leprosy and their biological consequences in an Avar Age (late 7th century CE) osteoarchaeological series of the Duna-Tisza Interfluve (Kiskundorozsma–Daruhalom-dűlő II, Hungary)
Source: PLoS One. 2022 Jun 23;17(6):e0265416. doi: 10.1371/journal.pone.0265416 (PMC9223331; doi:10.1371/journal.pone.0265416)
Supplement: Attachment — Submitted filename: response_to_reviewers.docx [file pone.0265416.s010.docx]

Dr JJ Cray Jr., PhD

Academic Editor

PLOS ONE

February 17, 2022

Dear Dr JJ Cray Jr.,

I am very thankful for the reviewers’ insightful and constructive comments regarding our manuscript entitled “The two extremes of Hansen’s disease – Different manifestations of leprosy and their biological consequences in an Avar Age (late 7^th^ century CE) osteoarchaeological series of the Duna-Tisza Interfluve (Kiskundorozsma–Daruhalom-dűlő II, Hungary)” that was submitted to the PLOS ONE (manuscript ID: PONE-D-21-21682). I am sure that the reviewers’ comments helped us to improve the quality of our manuscript. The main text, figures, and supplementary texts have been modified following the reviewers’ suggestions. The revised files have been uploaded to the submission site of the PLOS ONE.

**Responses to the suggestions:**

1) **Reviewer 3** commented that “*Overall, the paper requires some editing and wordsmithing. For example, the sentence on lines 34-36 would be better written as: “The led to severe deformation and disfigurement of the involved anatomical areas of the skeleton, resulting in the inability to perform the basic…..”. There are quite a few places through the ms where things could be stated more simply.*” Following **Reviewer 3**’s advice, the aforementioned sentence was amended. We also made all the corrections in the main text that **Reviewer 3** suggested in their review.

2) **Reviewer 3** noted that “*The authors also use the “hook” of stating that the cases they report are: (lines 42-43) “amongst the very few published cases with leprosy from the Avar Age of the Hungarian Duna-Tisza Interfluve” (which is repeated a number of times in the paper). I’m sure this is the case, but it seems overly narrow to me. Without knowing much about the temporal range or geographical location of the specimens, it doesn’t seem that significant to me. Could the authors qualify this? Does this represent a period of centuries across a wide geographical area that was known to have significant outbreaks of leprosy but these are really the few that are known? If the chronological range and geographical area is narrow and limited, this statement just doesn’t seem that important on a global scale.”* Unfortunately, we do not agree with **Reviewer 3** that “*if the chronological range and geographical area is narrow and limited”* the identification of leprosy cases from the Avar Age of the Duna-Tisza Interfluve (Hungary), like KD271 and KD520, cannot “*seem important on a global scale*”. As we already highlighted in our manuscript, *“(sub)genotyping of M. leprae aDNA, reconstructed from ancient human cases with Hansen’s disease, provides invaluable information about the origins and geographical distribution of leprosy bacilli, and the migration routes of their human host over time*” (lines 135–138 in the revised version of our manuscript). “*Recent subgenotypic data have revealed that Europe could be a key for the early spread and global dissemination of Hansen’s disease*” (lines 139–141 in the revised version of our manuscript). In addition, “*it is suggested that after the fall of the Roman Empire, the successive westward migration of the nomadic Avar tribes from Central Asia or Asia Minor via the Middle East led to the separate introduction or re-transmission of different M. leprae strains into Eastern and Central Europe (including Hungary) during the early mediaeval period*” (lines 147–151 in the revised version of our manuscript). We also highlighted in the S1 Text (entitled “*The Avar Khaganate in the Carpathian Basin between the 6^th^ and 8^th^ centuries CE*”) that “*the concentration of high-status burials lavishly furnished with weapons and gold artefacts in the Duna-Tisza Interfluve suggests that the Avar power centre lay in this region during the 6^th^ and 8^th^ centuries*.” Based on the above, this geographical area of Hungary was exceptionally important in the Avar Age (6^th^–8^th^ centuries CE), when leprosy could be introduced or re-transmitted to Europe (including Hungary). However, as we stated in our manuscript, the number of identified leprosy cases from the Duna-Tisza Interfluve of Hungary is very low – besides KD271 and KD520 that are discussed in our current paper, two other cases from the archaeological site of Hajós–Cifrahegy have been published in English (Marcsik et al. 2016 – reference 29 in the revised version of our manuscript). In addition, five other cases from the archaeological site of Kiskundorozsma–Daruhalom-dűlő II, two cases from Kiskundorozsma–Kettőshatár I, and one case from Kiskundorozsma–Kettőshatár II have been briefly described in a review by Marcsik and her colleagues in 2007 (reference 28 in the revised version of our manuscript). As these cases from the three Kiskundorozsma sites were published in Hungarian, unfortunately, the data are not available for the international readership. (It should also be noted that based on the re-evaluation of the aforementioned cases from the three Kiskundorozsma series, the diagnosis of leprosy is not that definitive in some of them.) Based on the above, we think that every identified prehistoric or historic leprosy case gives us a unique insight into the different manifestations of leprosy and their biological or even social consequences, and can extend our knowledge regarding the origins and geographical distribution of leprosy bacilli, and the migration routes of their human host over time. (As it was demonstrated by Mendum and colleagues in 2018 (reference 25 in the revised version of our manuscript), KD271 already did.) We hope that **Reviewer 3** will accept our argumentation.

3) **Reviewer 3** commented that “*Lines 50 – better to say “infectious disease found mainly in humans”. With that said, I’m curious, is it also transmissible among other primates?*”. Following **Reviewer 3**’s advice, the above-mentioned sentence was rephrased. As for **Reviewer 3**’s question regarding the transmission of leprosy among other primates, we have to note that unfortunately, we are not specialists in the transmission modes of leprosy among animals as we are palaeopathologists, so we do not know much about how leprosy may be transmitted among other primates. Nevertheless, we searched for information on the internet and found that it may be possible that the disease is transmissible among other primates: “*The finding of M. leprae-induced leprosy in wild chimpanzee populations raises the question of the origin(s) of these infections. Mycobacterium leprae is considered a human-adapted pathogen and previous cases of leprosy affecting wildlife were compatible with anthroponosis. Therefore, the prime hypothesis would be human-to-chimpanzee transmission. Potential routes of transmission include direct (such as skin-to-skin) contact and inhalation of respiratory droplets and/or fomites, with the assumption that, in all cases, prolonged and/or repeated exposure is required for transmission11. Chimpanzees at CNP are not habituated to humans and are not approached at distances that would allow for transmission via respiratory droplets. Although these chimpanzees inhabit an agroforest landscape and share access to natural and cultivated resources with humans28, present-day human–chimpanzee direct contact is uncommon. The exact nature of historic human–chimpanzee interactions at CNP remains, however, unknown. For example, robust data on whether chimpanzees were kept as ‘pets’ or were hunted for meat are lacking. Long-term human–chimpanzee coexistence in this shared landscape makes humans the most probable source of chimpanzee infection. However, multiple individuals from several chimpanzee communities across CNP show symptomatic leprosy demonstrating that M. leprae is now probably transmitted between individuals within this population* (Hockings et al. 2021)*.*” And “*To the best of our knowledge, this is the first paper to report the genomes of nonhuman primate M. leprae strains. While previous studies have shown that M. leprae strains can be transmitted to nonhuman primates, we did not know if naturally occurring leprosy in nonhuman primate was due to incidental infections by human M. leprae strains or by M. leprae strains specific to nonhuman primates. Our results suggest that nonhuman primates, such as chimpanzees and sooty mangabeys in Africa and cynomolgus macaques in Asia, may acquire M. leprae strains from humans as well as transmit these strains between themselves* (Honap et al. 2018).” We hope that **Reviewer 3** will be satisfied with our answer.

Hockings KJ, Mubemba B, Avanzi C, Pleh K, Düx A, Bersacola E, et al. Leprosy in wild chimpanzees. Nature 2021;598: 652–656. DOI: 10.1038/s41586-021-03968-4 (<https://www.nature.com/articles/s41586-021-03968-4#citeas>)

Honap TP, Pfister L-A, Housman G, Mills S, Tarara RP, Suzuki K, et al. *Mycobacterium leprae* genomes from naturally infected nonhuman primates. PLoS Negl Trop Dis 2018;12(1): e0006190. DOI: 10.1371/journal.pntd.0006190 (<https://journals.plos.org/plosntds/article?id=10.1371/journal.pntd.0006190>)

4) **Reviewer 3** advised that “*Line 56 – delete “of” before “caused*”.” Following **Reviewer 3**’s suggestion, “of” was deleted in line 56.

5) **Reviewer 3** suggested that “*Line 72 – replace “hardly” with “minimally*”.” Following **Reviewer 3**’s advice, “hardy” was replaced with “minimally” in line 72.

6) **Reviewer 3** commented that “*P. 6 – I appreciate the historical background provided, but it would be helpful to provide a few more details about the transmission of leprosy to some other parts of the world to show just how pervasive, transmissible, and fearful it was to humans. A recent paper by Nelson et al. (2022) describe what is purported to be the earliest directly dated case in the Americas. There is also early evidence for the spread of leprosy to India (ca. 4000 BP) (see Robbins et al. 2009, 2013). These are just a few, but there are others that would be worth noting. In addition, given some misunderstandings about leprosy and how it could be contracted, as well as the desire to help those afflicted, there were many leprosaria established in remote or protected areas (individual islands in the Caribbean and Pacific are well-known examples) to sequester and render aid*.” **Reviewer 3** also provided some references: 1) *Nelson, G.C., Dodrill, T.N. and Fitzpatrick, S.M., 2022. A probable case of leprosy from colonial period St. Vincent and the Grenadines, Southeastern Caribbean. International Journal of Paleopathology, 36, pp.7-13*.; 2) *Robbins, G., Tripathy, V.M., Misra, V.N., Mohanty, R.K., Shinde, V.S., Gray, K.M. and Schug, M.D., 2009. Ancient skeletal evidence for leprosy in India (2000 BC). PloS one, 4(5), p.e5669.*; and 3) *Robbins Schug, G., Blevins, K.E., Cox, B., Gray, K. and Mushrif-Tripathy, V., 2013. Infection, disease, and biosocial processes at the end of the Indus Civilization. PLoS One, 8(12), p.e84814*. We agree with **Reviewer 3** that the transmission routes of leprosy to parts of the world is a very interesting topic. However, we think that its detailed discussion would fit better in a review article not our current paper, as it focuses on a particular geographical region (Duna-Tisza Interfluve, Hungary) and time period (Avar Age). As **Reviewer 3** mentioned, we already provided the relevant historical background regarding the Avar Age of the Duna-Tisza Interfluve (Hungary) in our manuscript (main text and S1 Text). We are also not sure that it would be suitable to include a detailed discussion on the establishment of leprosaria into our current paper, as in the Avar Age of Hungary, no leprosaria have been established (the first leprosaria of the country were established centuries later). It should also be noted that in another paper from our research group (Spekker O, Tihanyi B, Kis L, Szalontai Cs, Vida T, Pálfi Gy, Marcsik A, Molnár E. 2022. Life and death of a leprosy sufferer from the 8th-century-CE cemetery of Kiskundorozsma–Kettőshatár I (Duna-Tisza Interfluve, Hungary) – Biological and social consequences of having Hansen’s disease in a late Avar Age population from Hungary. PLOS ONE. DOI: 10.1371/journal.pone.0264286), that will be published online in the PLOS ONE in a few days, the case we demonstrated illustrates the social attitude toward leprosy sufferers in early mediaeval Hungary – the social consequences of living and dying with leprosy in the late Avar Age community of Kiskundorozsma–Kettőshatár I, which is also located in the Duna-Tisza Interfluve (Hungary). In this paper, we briefly discuss that our present findings are in accordance with the results of previous studies on other Avar Age cemeteries from the present-day territory of Hungary, indicating that prior to the 13^th^ century CE, lepers have not been segregated from the healthy population, at least in death. To further highlight the social attitude toward leprosy sufferers in early medieval Hungary and to compare it to other time periods (e.g. periods after the 13^th^ century CE, when leprosaria were already established in Hungary), our research group is working on a review article about all the leprosy cases from the Avar Age of Hungary. During the preparation of our aforementioned manuscript, the references **Reviewer 3** provided us will be very useful. In summary, we hope that **Reviewer 3** will accept our argumentation. We really appreciate the references **Reviewer 3** provided us and for sure, will use them in our review article we are working on right now.

7) **Reviewer 3** advised that “*Line 215 – replace “of” with “in biological anthropology*”.” Following **Reviewer 3**’s suggestion, “of” was replaced with “in” in line 215.

8) **Reviewer 3** suggested that “*Lines 217-218 – use semicolons to separate sequentially numbered orders*”. Following **Reviewer 3**’s advice, semicolons were used to separate the sequentially numbered orders in lines 217–218.

9) **Reviewer 3** noted that “*Line 360 – typo - should be “With” (not “Whit”)*”. Following **Reviewer 3**’s comment, the typo was corrected in line 360.

10) **Reviewer 3** suggested that “*Line 636 – consider revising to: “It is important to note that apart from HD….”*.” Following **Reviewer 3**’s advice, the aforementioned sentence was rephrased in line 636.

11) **Reviewer 3** mentioned that “*Line 639 and in several places after (e.g., Lines 670-674). There are a number of cases where longer or multiple sentences are in parentheses. These should usually be reserved for shorter explanations, so consider incorporating these into the text without parentheses.*” Following **Reviewer 3**’s comment, the relevant parentheses were removed and the sentences were incorporated into the text throughout the main document.

12) **Reviewer 3** commented that “*Line 674 – replace “decided” with “determined*”.” Following **Reviewer 3**’s suggestion, “decided” was replaced with “determined” in line 674.

13) **Reviewer 3** suggested that “*Line 699 – remove double parentheses; instead, parentheses within parentheses should be changed to brackets (e.g., “….arthritis [RA] and psoriatic arthritis [PsA])*”. As **Reviewer 4** asked us to remove all the abbreviations, there are no double parentheses in the revised version of our manuscript; thus, there is no need to use brackets in the above-mentioned sentence in line 699.

14) **Reviewer 3** mentioned that “*Line 738 – this is not an adage exclusive of aDNA, but is a commonly used phrase in science and beyond*”. Based on **Reviewer 3**’s comment, the relevant sentence was amended in line 738.

15) **Reviewer 3** suggested that “*Line 745 – better to say “….in standing and walking and various physical actitivies.*”. In the aforementioned sentence, we wanted to highlight that KD520’s difficulties in standing and walking affected her everyday life, i.e., her ability to conduct domestic and occupational physical activities. In our opinion, if we would change the sentence how **Reviewer 3** suggested, its meaning would be changed, as well. Therefore, if **Reviewer 3** agrees, we would like to keep the sentence in its present form.

16) **Reviewer 3** mentioned that “*Figure 1 is insufficient for an international audience. It is unclear where this region is located on a regional or global map. Please include one that shows where “A” is found. Map also needs a scale and north arrow*.” As it was already indicated in the legend of Figure 1 (“*Fig 1. A) Map of Hungary showing the location of the Kiskundorozsma–Daruhalom-dűlő II archaeological site; B) Aerial photo of the Kiskundorozsma–Daruhalom-dűlő II archaeological site; and C) Plan drawing of the Avar Age cemetery of Kiskundorozsma–Daruhalom-dűlő II with the location of the burials of KD271 and KD520. (Contains information from OpenStreetMap and OpenStreetMap Foundation, which is made available under the Open Database License.)*”), Figure 1A presents not a region in Hungary but the map of the whole country, and illustrates where the Kiskundorozsma–Daruhalom-dűlő II cemetery, from which the two examined skeletons derive, is located within the country (green dot). In the originally submitted version of our manuscript, there was another map of Hungary and a small map of Europe at the upper left corner in Figure 1A, so the reader could easier figure out the location of Hungary in the continent. However, because of copyright issues, we had to remove this map of Hungary. It was very difficult for us to find another map of Hungary that fits all the copyright requirements of the PLOS ONE – finally, we decided to use the map that was included to the revised version as Figure 1A, as in our opinion, it is suitable to illustrate the location of the Kiskundorozsma–Daruhalom-dűlő II cemetery within Hungary. If **Reviewer 3** agrees, we would not like to change the map (Figure 1A) again, as it fits all the requirements of the PLOS ONE regarding maps/satellite images. We know that Hungary is a little country but we hope that most readers of the PLOS ONE know its location in Europe or the globe (and even if not, it can easily and quickly be checked on the internet). Following **Reviewer 3**’s advice, a scale and an arrow indicating north were put on the map of Hungary (Figure 1A).

17) **Reviewer 3** commented that “*Figures 2 and others – remove parentheses after letter, which is unnecessary*”. Following **Reviewer 3**’s suggestion, parentheses were removed after the letters in all figures.

18) **Reviewer 4** suggested that “*In the line 185 "prevention" should be "salvage*.". Following **Reviewer 4**’s advice, “preventive” was changed to “salvage” in line 185.

19) **Reviewer 4** commented that “*In lines 275, 288 and 290 the term "turbinate" is used incorrectly. In humans the inferior concha is a separate bone, different from animal "turbinate" bones. A basic anatomical terminology error. Use inferior nasal concha*.” Following **Reviewer 4**’s suggestion, “inferior turbinate bone” was changed to “inferior nasal concha” throughout the main text and relevant supplementary text (S2 Text).

20) **Reviewer 4** mentioned that “*In the era of electronic (digital, on-line) publications, the use of abbreviations/acronyms saving the cost of printing ink and paper for numerous copies of printed papers saves nothig while it appears to be pretentiously "scientific" and makes reading of manuscripts difficult. I recommend to use full terms instead of pretentious acronymes like TT, LL, HD etc*.” Following **Reviewer 4**’s advice, abbreviations were removed and their fully-written forms were used throughout the main text and supplementary texts.

In the revised version of our manuscript, we tried to execute all suggestions of the reviewers. I hope this new version will be suitable for publication in the PLOS ONE.

Thank you again for the reviewers’ insightful and constructive comments and your editorial work!

Yours sincerely,

Dr Olga Spekker, PhD

Postdoctoral researcher

Department of Biological Anthropology

University of Szeged

Közép fasor 52, H-6726 Szeged, Hungary

Email: [olga.spekker@gmail.com](mailto:olga.spekker@gmail.com)

Tel: +36 20 807 72 94
